# Orienting and Emotional Perception: Facilitation, Attenuation, and Interference

**DOI:** 10.3389/fpsyg.2012.00493

**Published:** 2012-11-16

**Authors:** Margaret M. Bradley, Andreas Keil, Peter J. Lang

**Affiliations:** ^1^Center for the study of Emotion and Attention, University of FloridaGainesville, FL, USA

**Keywords:** emotion, attention, orienting, psychophysiology, interference, competition

## Abstract

Human emotions are considered here to be founded on motivational circuits in the brain that evolved to protect (defensive) and sustain (appetitive) the life of individuals and species. These circuits are phylogenetically old, shared among mammals, and involve the activation of both subcortical and cortical structures that mediate attention, perception, and action. Circuit activation begins with a feature-match between a cue and an existing representation in memory that has motivational significance. Subsequent processes include rapid cue-directed orienting, information gathering, and action selection – What is it? Where is it? What to do? In our studies of emotional perception, we have found that measures that index orienting to emotional cues generally show enhanced circuit activation and response facilitation, relative to orienting indicators occasioned by affectively neutral cues, whether presented concurrently or independently. Here, we discuss these findings, considering both physiological reflex and brain measures as they are modulated during orienting and emotional perception.

## Introduction

Cues that signal danger or reward reflexively activate fundamental motivational circuits prompting heightened orienting and attention, facilitating the selection and implementation of appropriate action (Lang et al., 1997). In studies of emotion, therefore, many of the dependent measures that are regularly monitored, measured, and modulated are those traditionally considered to be components of the orienting response. It was Pavlov who first defined the “investigatory reaction” as a reliable behavioral pattern in which animals oriented receptors (ears, eyes, etc.) toward any novel stimulation (Pavlov, 1927). Later variously called the “novelty reflex,” the “exploratory reflex,” or the “orienting reflex” (or reaction) – this “*what is it*” response facilitated perceptual identification of a new, unexpected cue. Sokolov (1963) later reformulated the concept as a neuronal model in which orienting was a neurophysiological reaction to changes in the perceptual array. In this broader view, novel stimuli induce physiological changes in autonomic, somatic, and central systems that he collectively called the orienting response.

Subsequent research has confirmed that novel stimuli prompt orienting and that reactivity is heightened if cues are made “significant” through task-relevance and/or instructions. A number of researchers noted, however, that emotional cues seemed to elicit more pronounced orienting even in the absence of specific tasks or instructions (Bernstein, 1979; Maltzman, 1979; O’Gorman, 1979). These cues intrinsically activate motivational circuits, prompting what has been dubbed “*natural selective attention*” (Bradley, 2009). Thus, traditional measures of orienting, such as skin conductance, heart rate deceleration, pupil dilation, and modulation of the brain’s electrical activity are reliably enhanced in emotional perception. Pictures are particularly effective cues in activating motivational circuits, as these visual stimuli share many of the sensory and perceptual features with the actual object. This is vividly illustrated in phobic individuals, for whom a picture of a snake can prompt strong physiological reactions (Globisch et al., 1999; Ohman and Mineka, 2001; Sabatinelli et al., 2001) that mirror an actual encounter.

From a methodological viewpoint, the cues in a perceptual array at any moment can be roughly characterized as (1) cues that activate defensive and appetitive motivational circuits (i.e., “emotional” stimuli), and therefore are intrinsic targets of focal processing, and (2) cues that do not have strong, intrinsic motivational associations (i.e., “neutral” stimuli) that are not intrinsically focal and have not been made targets for focal processing on the basis of conditioning, task relatedness, or simple instruction. Functionally, then, the perceptual arrays used in emotion studies can include concurrent presentation of any of these stimulus types, and one or more cues may be the focus of measurement. In this brief review, we report data from our studies of picture perception which illustrate that measures that index orienting to emotional cues are reliably enhanced when presented in the context of neutral cues, whether focal or not; and that neutral cues (task-focal or simply concurrent) tend to show a diminished orienting response when presented in the context of emotional cues.

## Oculomotor Orienting

As Pavlov noted, orienting is associated with behavioral adjustments that direct relevant sense organs toward focal cues. In visual perception, eye tracking studies have shown that emotional cues systematically modulate the motor control of this receptor organ: focal processing of emotional pictures prompts a significantly greater number of fixations than processing neutral images, and this pattern is found regardless of whether pictures depict simple figure-ground compositions or perceptually more complex scenes (Bradley et al., 2011). Moreover, affectively engaging cues are scanned more broadly, with larger distance between successive fixations, which again is found regardless of perceptual complexity. When an emotional picture and a neutral picture are presented together in a perceptual array, affective cues draw a greater number of fixations, resulting in overall longer viewing duration; when multiple emotional cues comprise the perceptual array, those that are rated as most arousing (e.g., erotica, violence) draw a great number of fixations that are of longer duration (Bradley et al., unpublished manuscript). Overall, emotionally arousing cues preferentially determine ocular movements, affecting both the duration and breadth of information gathering, simultaneously reducing processing of neutral cues in the perceptual environment.

## Autonomic Nervous System Reactivity

**Figure 1:**
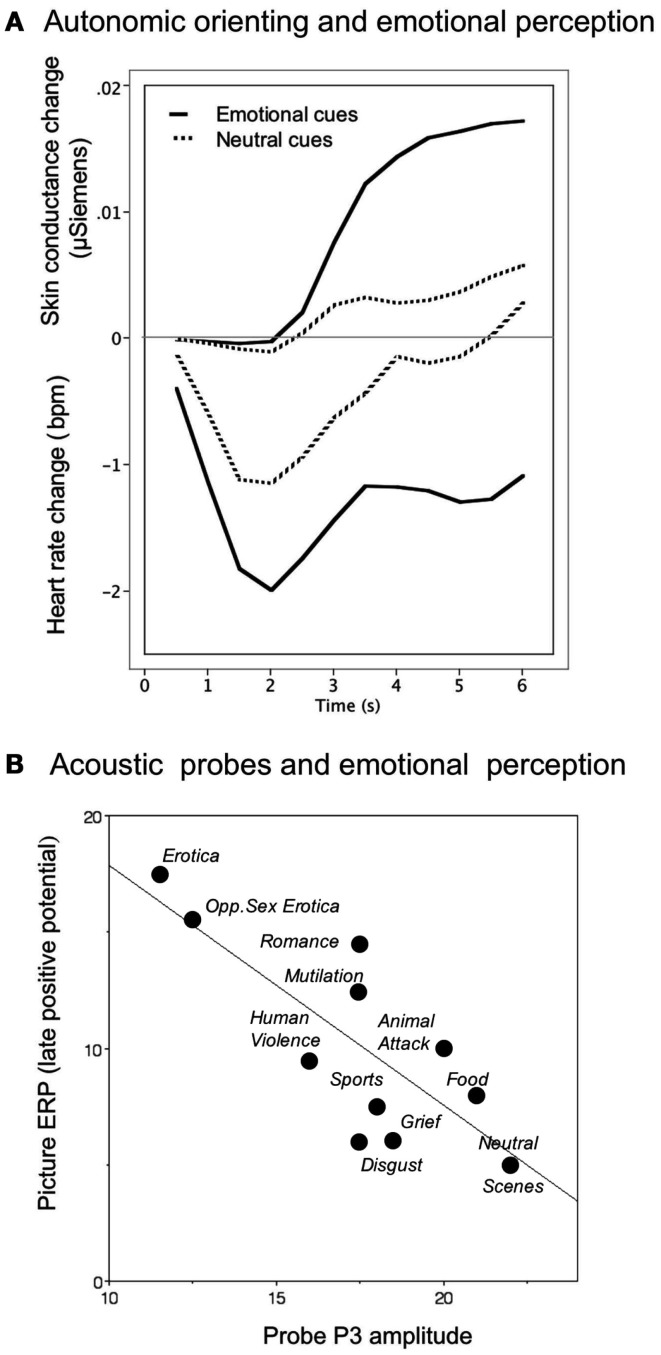
**(A)** Temporal dynamics of orienting in heart rate and skin conductance during emotional perception, with heightened cardiac deceleration and electrodermal reactivity when viewing emotional, compared to neutral, pictures. **(B)** The amplitude of the P3 component elicited by the presentation of an irrelevant acoustic probe during picture perception is inversely related to the amplitude of the late positive potential elicited by the picture itself. Larger late positive potentials, indicative of greater resource allocation to emotionally arousing cues, is associated with less resource allocation to the probe, resulting in smaller probe P3s.

Increase in palmar and plantar sweat gland activity is a classic physiological component of the orienting response (Sokolov, 1963; Maltzman, 1979). When measured via changes in the electrical resistance of the skin of the palm, even neutral pictures, when novel, prompt modest conductance increases, as illustrated in Figure 1A (Bradley, 2009). However, electrodermal changes are significantly elevated when viewing novel pleasant or unpleasant neutral pictures (Figure 1A; Lang et al., 1993). Moreover, pupil dilation shows a similar pattern: following a brief, obligatory pupil constriction associated with the light reflex, pupil diameter during orienting shows significantly greater dilation when viewing pleasant or unpleasant, compared to neutral, pictures (Bradley et al., 2008). For both pupil dilation and skin conductance activity, orienting reactions are largest for the most emotionally arousing cues.

The close relationship between orienting responses in pupil diameter and skin conductance is consistent with the fact that both are mediated by sympathetic nervous system activity (Bradley et al., 2008). In the view presented here, these autonomic reactions begin when a motivational cue is processed in visual cortex and activates the basolateral and then the central nucleus of the amygdala, which subsequently projects to the lateral hypothalamus, prompting broad engagement along the sympathetic chain, including neural connections to the rapidly dilating pupil, as well as to the more slowly responding palmar sweat glands. One function of sympathetic nervous system activity is to prepare the organism for potential action.

A classic measure of orienting is heart rate deceleration (Graham and Clifton, 1966; Lang et al., 1997), mediated by the parasympathetic branch of the autonomic nervous system (ANS). Heart rate orienting is characterized by a rapid, brief deceleration to any novel stimulus. When emotional pictures are presented, however, the deceleration can be deep and prolonged (see Figure 1A), particularly when viewing aversive stimuli. Although the sympathetic and parasympathetic branches of the ANS were once thought to be wholly reciprocal, it is now understood that they can be co-active depending on the stimulus context (Berntson et al., 1994). This is clearly seen during orienting, which involves motor inhibition (other than that necessary for directing sense organs) accompanied by heart rate deceleration. Because cardiac deceleration is quickly eliminated when the same picture is re-presented, it may reflect the sensory intake and information gathering function of orienting to novel or significant cues (Bradley, 2009).

When a cue indicates the necessity of immediate action, on the other hand, cardiac deceleration reverses and overall sympathetic cardiac control is asserted, prompting acceleration. In a recent experiment (Low et al., 2008), picture cues were presented sequentially, signaling increasingly imminent receipt of reward or loss that was dependent on the participant’s response time. In this perceptual context, skin conductance increased progressively in tandem with progressively decreasing heart rate until the penultimate signal for action. At that point, deceleration was replaced by cardiac tachycardia and a yet steeper increase in conductance until the action was accomplished. Taken together, then, enhanced orienting can be associated with either increased or decreased reactivity, depending upon the specific measure of orienting that is monitored.

## The Late Positive Potential

A number of different event-related potentials (ERP) have been linked to orienting and attention, including the P300 (P3), processing negativity, and a late positive complex (Ruchkin and Sutton, 1983; Donchin et al., 1984). An early central index of orienting, called the “orienting” wave (or “O-wave”) showed a slow positive change over central-parietal sensors when processing novel or significant stimuli (Connor and Lang, 1969; Loveless and Sanford, 1974; Rohrbaugh and Gaillard, 1983). A similar slow potential that shows enhanced positivity over centro-parietal sensors is the most reliable ERP modulated by hedonic content when emotional pictures are focal cues (e.g., Palomba et al., 1997; Cuthbert et al., 2000). This late positive potential is most enhanced for pictures rated highest in emotional arousal, regardless of whether they depict appetitive (e.g., erotica) or aversive (e.g., violence) hedonic content (Schupp et al., 2004). Directing attention to a focal cue through task-relevance, whether emotional or neutral, prompts a significantly enhanced late positive potential (Ferrari et al., 2008). Unlike the cardiac component of the orienting response, the late positive potential continues to be enhanced when viewing emotional, compared to neutral, pictures, even following multiple contiguous repetitions (Ferrari et al., 2011), suggesting that this component of the orienting response may index motivational activation, which does not change with mere stimulus repetition (Bradley, 2009).

## Probing Perceptual Orienting

In addition to measures which directly reflect enhanced orienting to emotional cues, perceptual processing can be probed by presenting brief, discrete acoustic stimuli (e.g., tones or noise bursts) during picture viewing, and measuring a variety of orienting responses to the secondary probe stimulus. One electrophysiological index of orienting is the amplitude of a centro-parietal P3 component: when an emotional (pleasant or unpleasant), compared to neutral, picture is the focal cue, the amplitude of the P3 elicited by the acoustic probe is attenuated, suggesting reduced orienting to concurrent cues presented during emotional perception (Schupp et al., 1997). Interestingly, the same pattern of reduced P3 amplitude is found when acoustic probes are presented during perception of pleasant and unpleasant environmental sounds (Keil et al., 2007), suggesting that attenuated orienting to secondary cues is not restricted to cross-modal contexts.

Another measure of probe processing – speeded reaction time in a simple detection task – is also consistent with attenuated perceptual processing of the probe during emotional perception. The speed of concurrent probe detection is significantly slower when viewing emotional, compared to neutral, pictures (Bradley et al., 1999), particularly immediately after picture onset, when orienting is maximal. For the reaction time measure, differences in speed of responding disappear about 1 s after picture onset, whereas, interestingly, probe P3 amplitude continues to discriminate between processing of emotional and neutral pictures for up to 4 s following picture onset (Bradley et al., 2008), suggesting that these measures of probe processing reflect different facets of phasic and sustained orienting activity.

When probe P3 amplitude is assessed as it varies with the late positive potential that indexes orienting to the focal picture, an inverse relationship is found as illustrated in Figure 1B, with larger LPPs associated with smaller probe P3s, indicative of a trade-off in which heightened attention to the picture results in fewer resources available for processing the probe. Unlike the late positive potential, however, which continues to be modulated following picture repetition, probe P3 amplitude is no longer different when viewing emotional, compared to neutral, pictures after multiple contiguous repetitions (Ferrari et al., 2011), suggesting that this component of the orienting response may index resource allocation, which is expected to diminish with stimulus repetition.

## Steady-State Visual Evoked Potentials: Processing Focal Cues

Perception of motivationally relevant cues is reliably associated with heightened activity of the visual cortex (Desimone, 1996). Both functional neuroimaging (e.g., Kastner and Ungerleider, 2000) and animal studies of selective attention (e.g., Reynolds et al., 1999) have garnered empirical support for this hypothesis by studying visual responses to significant stimuli in both humans and animals. The visual sensory response to a perceptual cue such as a picture can be easily measured using the ssVEP, a continuous oscillatory brain response elicited by a visual stimulus which, when rapidly brightness-modulated (flickered), prompts electrical activity at the same frequency as the flickered stimulus. The ssVEP can be measured in the EEG by sensors placed over the occipital cortex, and the evoked neural activity subsequently precisely defined in the frequency and time-frequency domains.

**Figure 2:**
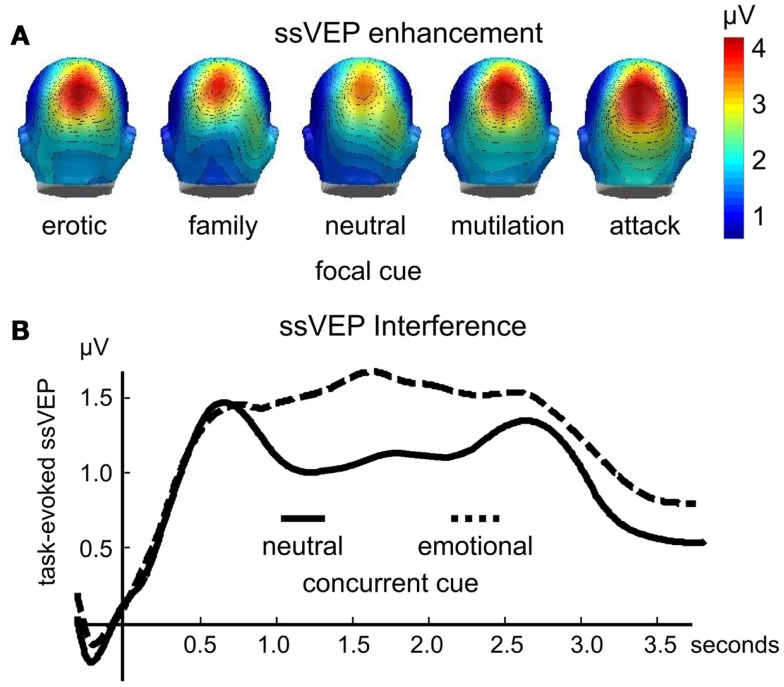
**The amplitude of the time varying steady-state visual evoked potential is enhanced when evoked by focal emotional, compared to neutral pictures [(A); redrawn from data in Keil et al., 2008]**. Conversely **(B)**, the ssVEP is reduced when evoked by a focal task stimulus that competes with an emotionally engaging distractor (data from Wieser et al., 2012). This interference lasts between several hundreds of milliseconds and several seconds, depending on the complexity and strength of the distractor and the primary task (Müller et al., 2008).

Enhanced ssVEP amplitudes to visual stimuli are reliably observed as a function of instructed attention (Muller et al., 2006). However, as with other measures of heightened attention and orienting, the ssVEP elicited by flickering emotional pictures is heightened in amplitude in the absence of instructions or task-relevance. In a first study (Keil et al., 2008), participants simply viewed pictures flickering at a rate of 10 Hz, which included emotional cues that depicted pictures of erotic couples, families, mutilated bodies, and attack scenes, as well as pictures of everyday events and objects. The amplitude of the ssVEP signal was reliably enhanced when processing emotional, compared to neutral, pictures over both occipital, and parietal electrode sites (see Figure 2A).

To the extent that the ssVEP is generated in extended visual cortex, these findings may be taken as evidence for sensory facilitation when a stimulus engages motivational systems, in line with a number of fMRI studies showing heightened activation in visual cortex for motivationally relevant cues (Lang et al., 1998; Bradley et al., 2003). Accordingly, the extent of activity in visual cortex during picture viewing is explained to a large extent by intensity of emotional arousal, measured either by ratings of affect or by the skin conductance orienting response (Keil et al., 2008). When examined using advanced time-series statistics (Keil et al., 2009), the spatio-temporal information inherent in the ssVEP points to recurrent, bi-directional information flow from anterior brain areas back into visual cortex, suggesting widespread orienting activity.

Supporting the idea that sensory facilitation for emotionally engaging cues arises as a consequence of recurrent information flow between sensory areas and motivational circuits (Lang, 1979; Sabatinelli et al., 2009), the ssVEP amplitude is also modulated following classical aversive conditioning. Thus, perceptual processing of a neutral CS+ (the conditioned stimulus predicting the aversive unconditioned stimulus) is enhanced, compared to the CS− following contiguous pairing (Moratti and Keil, 2005). Across studies, results show that the enhanced sensory ssVEP for the CS+ parallels the development of broader defense activation, indexed by augmented heart rate orienting and skin conductance increase (Moratti et al., 2006).

## Steady-State Visual Evoked Potentials: Competition

One advantage of the steady-state potential technique is that it allows one to tag different cues in the perceptual array by flickering them at different rates. Such frequency tagging yields separable signals even for stimuli that spatially overlap. In addition, time-frequency analyses can describe the time course of perceptual processing to a specific focal or secondary cue in a perceptual array, assessing facilitation, or interference (Wieser and Keil, 2011). Several studies have used this technique to assess effects of concurrent emotional “distractors” on visual processing of neutral cues. For instance, Müller et al. (2008) recorded ssVEPs in response to clouds of randomly moving flickering dots (i.e., random dot kinematograms) in which observers were instructed to detect instances of non-random, coherent movement in a subset of dots. These kinematograms were superimposed on pleasant, unpleasant, neutral, or scrambled pictures, and participants were instructed that the pictures were irrelevant to the focal task. The amplitude of the ssVEP elicited by the dots was reduced for extended periods of several hundreds of milliseconds, when distractors were pleasant and unpleasant pictures, compared to neutral or scrambled pictures (see Figure 2B).

In a related study, participants performed a foreground task that required detection of pattern changes in a black-and-white grating that was spatially superimposed on emotional or neutral pictures. The grating and the pictures were tagged at different frequencies (Wieser et al., 2012). Again, the ssVEP evoked by the neutral, task-relevant Gabor grating was reliably diminished specifically for affectively engaging pictures, as illustrated in Figure 1B. Both studies converged in terms of the time course of these distractor effects, showing a decrease in both target detection accuracy and ssVEP amplitude diminution that lasted for approximately 800 ms after stimulus onset (see Figure 1B). Suppressive or interfering effects of an emotionally engaging distractor also often affect subsequently presented stimuli. Behaviorally, this is evident in RSVP paradigms, in which detection of a neutral target is impaired when it appears between 200 and 800 ms after a task-irrelevant emotional distractor (Most et al., 2005; Ihssen et al., 2007).

## Endnote

Taken together, then, a number of the traditional measures used as evidence of affective engagement in studies of emotional perception index the heightened orienting and attention that we suggest reflects activation of defensive or appetitive motivational systems that have evolved to support survival. Adjustment of sensory receptors, enhanced resource allocation, and dedicated perceptual processing are initiated in the service of stimulus identification and preparation for action. Here, we suggest that a critical variable determining whether emotion facilitates or inhibits orienting in emotional perception is the selected dependent measure: measures that index orienting to emotional cues tend to show enhancement and facilitation when these cues are present in the perceptual array – whether as intrinsically focal cues or as concurrent “distractors.” On the other hand, orienting indicators occasioned by affectively neutral cues, whether presented concurrently or independently, tend to show attenuation and interference.

Orienting is not a unitary response, and the rate at which specific components change, as well as their timing, often varies with the dependent measure under investigation. Thus, modulation by emotion can be phasic or sustained, immediate or delayed, presumably reflecting the specific function of different components of the orienting response (Bradley, 2009), and adding complexity in understanding effects of emotion during perception. Differential effects of stimulus repetition on specific components of the orienting response provide information which can assist in inferring their function as information intake, vigilance, resource allocation, preparation for action, and other processes mediating perception and action (Bradley, 2009). Importantly, orienting is primarily a hallmark of emotional perception, and emotional modulation of higher-level cognitive domains, such as memory, decision-making, and regulatory processes will likely have different dynamics.

From a cognitive viewpoint, the data presented here are consistent with the conceptualization that perceptual processing draws on a limited pool of shared resources (Deutsch and Deutsch, 1963) in which cues that activate motivational systems naturally utilize more resources, resulting in measurable trade-offs for concurrent cues. From a neurophysiological perspective, the same reciprocal pattern is often conceptualized as reflecting competition in dedicated neural circuits (Shafer et al., 2012), in which cues of high motivational relevance have priority. Although stated at different levels of analysis and using different semantic terms, both accounts are consistent with the hypothesis that emotional cues capture attention, based on their evolutionary significance in the battle for survival, and that perceptual processing of concurrent non-motivationally relevant tends to suffer when competing with emotionally significant stimuli.

## Conflict of Interest Statement

The authors declare that the research was conducted in the absence of any commercial or financial relationships that could be construed as a potential conflict of interest.
